# Spontaneous isolated gastric intramural hematoma combined with spontaneous superior mesenteric artery intermural hematoma: a rare case

**DOI:** 10.1186/s12877-024-04991-6

**Published:** 2024-04-23

**Authors:** Zhenxing Zhang, Shan Wang, Kelong Tao, Guolin Zhang, Danling Guo, Yu Zhang, Guangen Xu

**Affiliations:** 1 Department of Gastrointestinal Surgery, Shaoxing People’s Hospital, Shaoxing, 312000 China; 2 Department of Special Examination, Shaoxing People’s Hospital, Shaoxing, 312000 China; 3 Department of Radiology, Shaoxing People’s Hospital, Shaoxing, 312000 China

**Keywords:** Gastric hematoma, Gastric intramural hematoma, Superior mesenteric artery intermural hematoma

## Abstract

**Background:**

Gastric intramural hematoma is a rare disease. Here we report a case of spontaneous isolated gastric intramural hematoma combined with spontaneous superior mesenteric artery intermural hematoma.

**Case presentation:**

A 75-years-old man was admitted to our department with complaints of abdominal pain. He underwent a whole abdominal computed tomography (CT) scan in the emergency department, which showed extensive thickening of the gastric wall in the gastric body and sinus region with enlarged surrounding lymph nodes, localized thickening of the intestinal wall in the transverse colon, localized indistinct demarcation between the stomach and transverse colon, and a small amount of fluid accumulation in the abdominal cavity. Immediately afterwards, he was admitted to our department, and then we arranged a computed tomography with intravenously administered contrast agent showed a spontaneous isolated gastric intramural hematoma combined with spontaneous superior mesenteric artery intermural hematoma. Therefore, we treated him with anticoagulation and conservative observation. During his stay in the hospital, he was given low-molecular heparin by subcutaneous injection for anticoagulation therapy, and after discharge, he was given oral anticoagulation therapy with rivaroxaban. At the follow-up of more than 4 months, most of the intramural hematoma was absorbed and became significantly smaller, and the intermural hematoma of the superior mesenteric artery was basically absorbed, which also confirmed that the intramural mass was an intramural hematoma.

**Conclusion:**

A gastric intramural hematoma should be considered, when an intra-abdominal mass was found to be attached to the gastric wall. Proper recognition of gastric intramural hematoma can reduce the misdiagnosis rate of confusion with gastric cancer.

## Background

Gastric intramural hematoma is a rare disease. The exact incidence of gastric hematomas is unknown. Intramural hematomas of the gastrointestinal tract are well described in the esophagus and duodenum [1]. However, intramural hematomas in the stomach have been rarely reported [2–4]. The main causes of intramural hematomas in the stomach include anticoagulant therapy, coagulopathy, trauma, and aneurysm, as well as idiopathic or congenital (endoscopic treatment and surgery). Here we report a case of spontaneous isolated gastric intramural hematoma combined with spontaneous superior mesenteric artery intermural hematoma.

## Case presentation

A 75-year-old man was brought to our emergency department complaining of abdominal pain, predominantly in the upper abdomen and around the umbilicus. The pain started at 3 h and got progressively worse. Physical examination revealed with a blood pressure of 116/ 70 mmHg, heart rate of 60 beats/minute, oxygen saturation of 100% (on room air), and respiratory rate of 17/ minute. The signs of abdominal physical examination were negative except epigastric pressure pain. His past medical history included Parkinson's disease (PD), cerebral ischemia. He had no history of pancreatitis, abdominal trauma and other abdominal surgery except hemorrhoid surgery. He was taking aspirin, cytarabine sodium and medroxyprogesterone. His laboratory findings included erythrocytes count of 4.62*10^12^/L, hemoglobin level of 133 g/L, hematocrit level of 42.1%, prothrombin time of 11.4 s, activated prothrombin time of 22.2 s, fibrinogen level of 2.17 g/L and normal values of platelets and D-dimer, but a bleeding time was not performed. Blood chemistry laboratory tests were within normal limits. He underwent a whole abdominal CT scan (2022–10-27) in the emergency department, which showed extensive thickening of the gastric wall in the gastric body and sinus region with enlarged surrounding lymph nodes, localized thickening of the intestinal wall in the transverse colon, localized indistinct demarcation between the stomach and transverse colon, and a small amount of fluid accumulation in the abdominal cavity. Afterwards, he was admitted to our department with a diagnosis of gastric neoplasm.

Post-admission physical examination and gastroduodenoscop suggested no significant abnormality. But contrast-enhanced abdominal CT (2022–10-29) suggested hematoma formation on the lateral side of the gastric lesser curvature with perihepatic and abdominopelvic blood/fluid accumulation considered, and multiple peri-gastric lymph nodes enlarged. Interstitial hematoma of the superior mesenteric artery is considered. Computed tomography angiography of the abdominal aorta (2022–10-30) showed a filling defect in the superior mesenteric artery with luminal stenosis, suggesting a possible thrombosis of a vessel coming off the superior mesenteric artery. Concomitantly, hematoma formation on the lateral side of the gastric lesser curvature with perihepatic and abdominopelvic blood/fluid accumulation was considered, and multiple peri-gastric lymph nodes were enlarged. We did not conduct invasive treatment, such as endovascular management or surgery considering that no obvious bleeding site or any mass was found on enhanced abdominal CT and computed tomography angiography (CTA) of the abdominal aorta. Then we took conservative anticoagulation treatment for low-molecular heparin by subcutaneous injection. The patient recovered gradually after conservative anticoagulation treatment without signs of continuous bleeding or increased abdominal pain, and he was discharged after 5 days. Physical examination at the time of discharge suggested blood pressure of 121/63 mmHg, heart rate of 87 beats/minute. His laboratory tests at discharge included erythrocytes count of 3.53*10^12^/L, hemoglobin level of 104 g/L, hematocrit level of 33.3%. He was given oral anticoagulation therapy with rivaroxaban 20 mg quaque die after discharge.

**Fig. 1:**
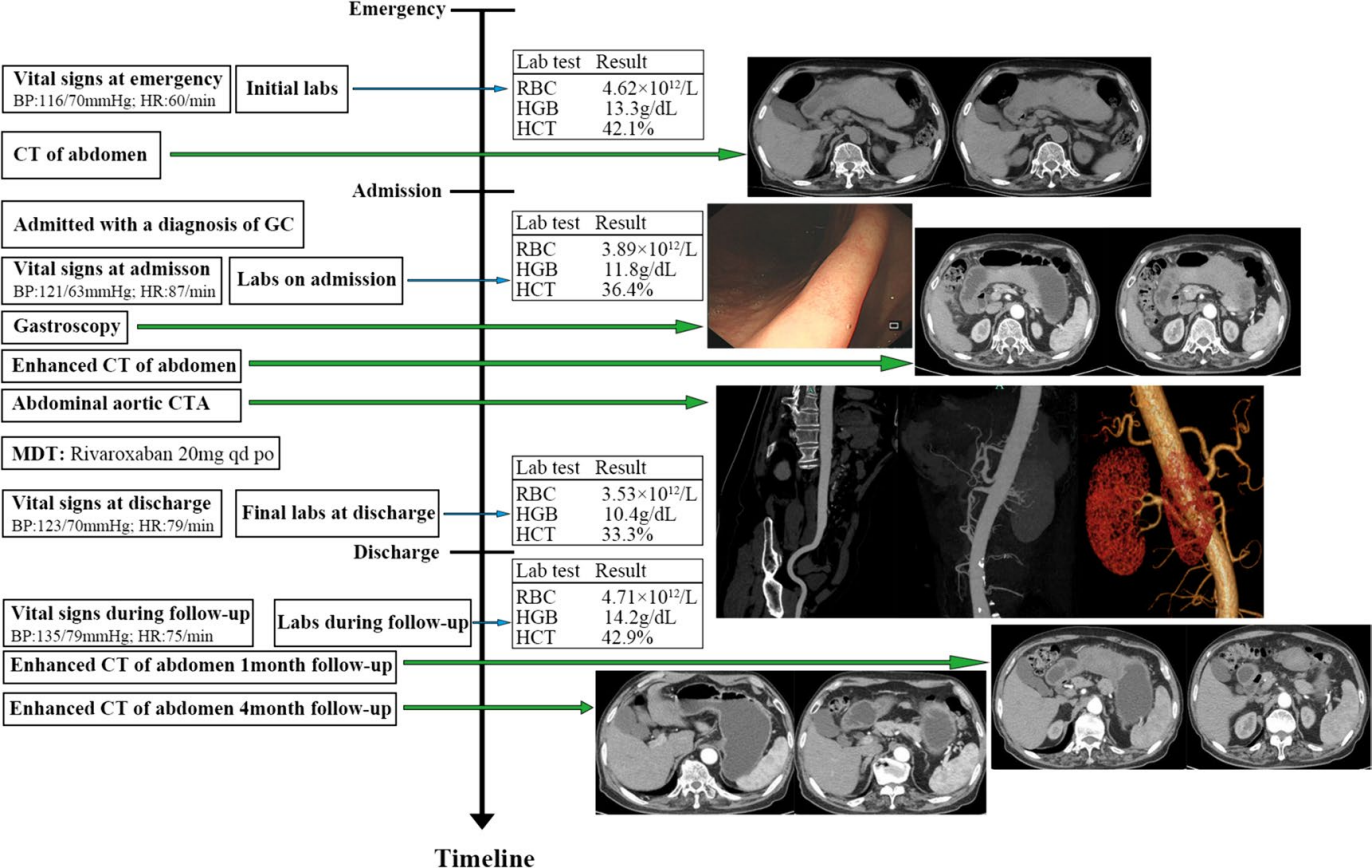
The timeline from emergency to follow-ups

We performed follow-ups for the patient regularly at the hospital outpatient department. At the follow-up about 1 month after discharge, the patient had no other discomfort such as abdominal pain. The results of the about 1-month follow-up after discharge showed blood pressure of 135/79 mmHg, heart rate of 75 beats/minute. His laboratory tests at discharge included erythrocytes count of 4.71*10^12^/L, hemoglobin level of 142 g/L, hematocrit level of 42.9%. Contrast-enhanced abdominal CT (2022–11-18) suggested the hematoma on the lateral side of the lesser curvature of the stomach is less absorbed than the imaging findings on 2022–10-29, the perihepatic blood/fluid accumulation is less than before, and the pelvic blood/fluid accumulation is essentially similar than before. Intermural hematoma of the superior mesenteric artery with localized entrapment changes. The contrast-enhanced abdominal CT (2023–03-10) of the about 4-month follow-up after discharge showed a small hematoma on the side of the gastric lesser curvature was mostly decreased and the intermural hematoma of the superior mesenteric artery was largely decreased and abdominopelvic blood/fluid accumulation disappeared compared to the imaging findings on 2022–11-18. The timeline from emergency to follow-ups is presented in Fig. 1.

## Discussion

Gastric intramural hematoma is rare with only a few cases reported to date [1–4]. Here we report the first case of which described a spontaneous isolated gastric intramural hematoma combined with spontaneous superior mesenteric artery intermural hematoma. Which is particularly rare.

Within the gastric wall there are a large number of arteries and veins distributed in a network and with a significant number of blood vessels in the submucosal layer [5]. When an artery is injured due to external factors, it can lead to the formation of a hematoma in the submucosal or muscular layer [6]. Spontaneous isolated intramural hematoma of the superior mesenteric artery is characterized by completely thrombosed false lumen which was often considered as a subset of spontaneous isolated dissection of the superior mesenteric artery (SIDSMA) and only included few cases in the current literature [7, 8].However, in our case, intramural hematoma of the superior mesenteric artery did not reveal obvious signs of dissection on CTA of the abdominal aorta, but rather suggested the presence of a thrombosis of a vessel coming off the superior mesenteric artery. In our case, this patient had an isolated intramural hematoma of the stomach and an isolated hematoma of the superior mesenteric artery, and there appeared to be no continuity between the two sites.

GIH usually arises secondary to another condition or intervention. The causes of this disease include anticoagulant medication [9], coagulopathy, ulcer disease, aneurysm, upper gastrointestinal endoscopy, procedure-related damage, or even in the presence of infection [10–15].

Spontaneous isolated intramural hematoma of the SMA is known as a variant form of SIDSMA, and factors likely affecting its nature are complex, including a history of connective tissue disease, fibromuscular dysplasia, atherosclerosis, and elastosis [16, 17]. This patient denied a history of connective tissue disease, lupus erythematosus, giant cell arthritis, mesenteric arthritis, Ehlers-Danlos disease, and it is important to monitor the subsequent literature on this aspect of autoimmune disease. The etiology of the gastric intramural hematoma and SIHSMA in this case is unknown. This patient had a long history of oral aspirin medication, but his coagulation function and platelets were not substantial abnormal, but a bleeding time was not performed. We therefore considered this case to be an idiopathic hematoma. It is important to distinguish it from various submucosal tumors including gastrointestinal mesenchymal tumors (GISTs) because of the submucosal tumor-like appearance of the hematoma [18]. In previously published case reports, there have been confusing misclassifications in the diagnosis of intramural hematomas in the stomach.

Symptomatic intramural gastric hematomas are extremely rare, and less than 15 cases underwent surgical treatment [19, 20], and of the cases treated with surgery, only a small percentage of cases had a correct preoperative diagnosis, with a preoperative diagnosis of gastric tumor, a preoperative diagnosis of pancreatic cyst, and a preoperative diagnosis of unknown [20]. In this case, the patient presented with acute abdominal pain and was diagnosed with a gastric tumor in an emergency abdominal (CT) scan, and a revised diagnosis of intramural hematoma was made after performing an enhanced abdominal CT in admission, which was confirmed in the subsequent anticoagulation follow-up treatment. Their treatment recommendations are not standardized due to their rarity [19]. Gastric intramural hematoma is treated in various ways, including surgery and transcatheter arterial embolization, endoscopic and percutaneous drainage, and conservative treatment [20]. In this case the patient successfully recovered with conservative anticoagulation.

## Conclusions

Here we reported a case of spontaneous isolated gastric intramural hematoma combined with spontaneous superior mesenteric artery intermural hematoma. A gastric intramural hematoma should be considered, when an intra-abdominal mass was found to be attached to the gastric wall. Proper recognition of gastric intramural hematoma can reduce the misdiagnosis rate of confusion with gastric neoplasm.

## Data Availability

All patient data and clinical images adopted are contained in the medical files of Shaoxing People’s Hospital. The data supporting the conclusions of this article are included within the article and its figures and tables.

## Abbreviations

CT: Computed tomography
CTA: Computed tomography angiography
SIDSMA: Spontaneous isolated dissection of the superior mesenteric artery
